# Dual Residual Denoising Autoencoder with Channel Attention Mechanism for Modulation of Signals

**DOI:** 10.3390/s23021023

**Published:** 2023-01-16

**Authors:** Ruifeng Duan, Ziyu Chen, Haiyan Zhang, Xu Wang, Wei Meng, Guodong Sun

**Affiliations:** 1 School of Information Science and Technology, Beijing Forestry University, Beijing 100083, China; 2 Engineering Research Center for Forestry-Oriented Intelligent Information Processing of National Forestry and Grassland Administration, Beijing 100083, China

**Keywords:** signal denoising, convolutional autoencoder, channel attention, residual connection, AWGN

## Abstract

Aiming to address the problems of the high bit error rate (BER) of demodulation or low classification accuracy of modulation signals with a low signal-to-noise ratio (SNR), we propose a double-residual denoising autoencoder method with a channel attention mechanism, referred to as DRdA-CA, to improve the SNR of modulation signals. The proposed DRdA-CA consists of an encoding module and a decoding module. A squeeze-and-excitation (SE) ResNet module containing one residual connection is modified and then introduced into the autoencoder as the channel attention mechanism, to better extract the characteristics of the modulation signals and reduce the computational complexity of the model. Moreover, the other residual connection is further added inside the encoding and decoding modules to optimize the network degradation problem, which is beneficial for fully exploiting the multi-level features of modulation signals and improving the reconstruction quality of the signal. The ablation experiments prove that both the improved SE module and dual residual connections in the proposed method play an important role in improving the denoising performance. The subsequent experimental results show that the proposed DRdA-CA significantly improves the SNR values of eight modulation types in the range of −12 dB to 8 dB. Especially for 16QAM and 64QAM, the SNR is improved by 8.38 dB and 8.27 dB on average, respectively. Compared to the DnCNN denoising method, the proposed DRdA-CA makes the average classification accuracy increase by 67.59∼74.94% over the entire SNR range. When it comes to the demodulation, compared with the RLS and the DnCNN denoising algorithms, the proposed denoising method reduces the BER of 16QAM by an average of 63.5% and 40.5%, and reduces the BER of 64QAM by an average of 46.7% and 18.6%. The above results show that the proposed DRdA-CA achieves the optimal noise reduction effect.

## 1. Introduction

The improvement of the SNR plays an important role in both the cooperative and non-cooperative fields of wireless communication systems. In cooperative communication, improving the modulation signal’s SNR can enhance the signal transmission quality and reduce the demodulation error rate. When it comes to non-cooperative communication, the improvement of the SNR is conducive to improving the modulation classification accuracy, and then signal interception or interference can be performed more effectively. Therefore, the denoising method of modulation signals has always been the research focus of wireless communication.

In the traditional signal denoising methods, Parolai et al. in Ref. [1] use the wavelet transform (WT) denoising method to deal with seismic wave signals, eliminate noise coefficients below the threshold value, and then reconstruct signals through inverse WT. However, it is difficult for the WT denoising method to select the proper parameters, including wavelet base, threshold, and decomposition layers. Milani et al. [2] use the least mean square (LMS) algorithm to eliminate the noise of acoustic signals, and Albu et al. [3] use the recursive least squares (RLS) algorithm for signal noise reduction. The RLS algorithm is an improvement on the LMS algorithm and it adopts recursive calculation and has better performance than the LMS adaptive transverse filter. But Haykin et al. [4] point out that the performance of the RLS algorithm is not stable due to its internal positive feedback mechanism. Li et al., in Refs. [5,6,7], use the principal component analysis (PCA) method to reduce the signal’s noise. The PCA algorithm recombines the original variables into a group of new irrelevant variables by reducing the dimension of the data, and extracts important characteristic variables according to the actual needs. However, it cannot accurately estimate the number of potential hidden variables in signals [8]. Moreover, the PCA method cannot analyze the principal components accurately when the noise’s energy is greater than the signal’s energy. Bekara et al. [9,10] adopt the singular value decomposition (SVD) method for noise reduction and achieve satisfactory results. The SVD algorithm is a generalization of spectral analysis theory on arbitrary matrices, but the selections of its effective rank and the reconstruction matrix have a great influence on the performance of the algorithm, and the matrix decomposition of the SVD algorithm is not highly interpretable. Zhang et al. [11] propose a noise reduction multi-carrier CDSK chaotic communication system based on Schmidt orthogonalization, and obtain good BER performance only under certain parameter conditions by using a sliding filter to reduce noise. It can be seen that there are still some critical issues in the traditional denoising methods that have not been well-addressed. Most of the methods require researchers to know the channel parameters and obtain the channel transmission characteristics by sending a training sequence, which leads to low transmission efficiency and poor channel utilization.

Recently, deep learning has not only achieved great success in the fields of computer vision and natural language processing, but has also provided a new solution for the noise reduction of modulation signals. Chang et al. [12] propose a convolutional neural network (CNN)-based hybrid cascade structure to replace the traditional equalizer in the communication system. Wada et al. [13] use two fully connected layers (FC) as denoising autoencoders to reduce noise of modulation signals. Zhao et al. [14] propose a deep neural network co-evolving simultaneously at two different scales to enhance the denoising ability of the model. Johanna et al. [15] use a CNN-based denoising model to suppress interference in real-world radar measurements. Hwanjin Kim et al. [16] develop and compare a machine learning (ML)-based channel predictor and a vector Kalman filter (VKF)-based channel predictor using the spatial channel model (SCM), which is widely used in the 3GPP standard.

Due to the limitations of the simple network model, the above five methods cannot extract deep signal features, resulting in limited SNR improvement. In deep convolutional networks, shallow convolution can extract detailed edge data, while deep convolution can selectively acquire useful semantic information. Therefore, deep convolutional networks are also widely used in signal denoising. In 2017, Zhang et al. [17] propose the residual learning of deep CNN for image denoising (DnCNN) and achieve excellent performance. In 2019, Khan et al. [18] extend the DnCNN method to the field of noise reduction for modulation signals, and experiments show that it has achieved a favorable denoising effect on high-order QAM modulation signals that are susceptible to noise interference. In the same year, Yin et al. [19] propose a full convolutional denoising autoencoder to reduce the noise of underwater acoustic signals, and obtain better results in both the time domain and frequency domain, compared with traditional methods. Xue et al. [20] design a wireless signal enhancement network based on the specialized Generative Adversarial Networks, which can adaptively learn the characteristics of signals and realize signal enhancement in time-varying systems. However, such methods ignore the correlation between the deep feature maps and the shallow feature maps, and they often have high training complexity because of deep layers. Refs. [21,22,23,24,25,26] consider the correlation between feature maps of different scales and depths in the model’s design, and the feature graphs of different levels are added by using residual connections [27], which effectively alleviates the difficulty of training deep network models and resolves the problems of gradient disappearance and explosion. The simple connections between feature maps of different scales and depths still make it difficult to selectively strengthen some important features, degrading the performance of the network model.

The channel attention mechanism is a very powerful method for optimizing network performance. It can selectively strengthen feature values of important channels by changing the weight of the channel, thus improving the expression ability of the network and achieving higher performance. Yancheng et al. [28] employ the residual encoder-decoder structure and multi-attention mechanism fusion module to perform feature map reuse and self-recalibrating features, which can significantly improve the performance of ultrasound image denoising. Li et al. [29] propose a new end-to-end semantic segmentation network, which integrates lightweight spatial and channel attention modules that can refine features adaptively. Zhou et al. [30] propose to incorporate an attention mechanism including a spatial attention module and a channel attention module into a U-Net architecture to re-weight the feature representation spatially and channel-wise to capture rich contextual relationships for better feature representation.

Based on the above analysis, in this paper, we adopt a deep convolutional network to construct a denoising autoencoder, in which more effective residual connections between different scales and depths are explored to extract richer features and optimize network degradation problem. We also try to integrate the squeeze-and-excitation (SE) module [31] into the network as a typical channel attention mechanism. The SE module can first obtain the global features of each channel in the denoising network through the global average pooling operation [32], and the global features reflect the correlations between and effectiveness of the channel feature maps. Then, the global features are weighted to make the channel with a strong correlation and high effectiveness have more weight, and vice versa. By selectively strengthening the channel weights and emphasizing the features of important channels, the SE module makes the network have a strong expression ability, leading to superior performance. The contributions of this paper can be summarized as follows.

(1) We choose the convolutional denoising autoencoder as the noise reduction model for modulation signals. Residual connections are added not only inside the encoding (decoding) block but also between the encoding (decoding) blocks of different depths to form a double residual connection.
(2) The SE module is improved and introduced into the denoising autoencoder to optimize the feature extraction of modulation signals. The improved SE module retains the advantages of selective enhancement for channel features, and the number of parameters is fewer, which makes the network model easier to train and achieve a better noise reduction effect.
(3) In the decoding module of the denoising autoencoder, transpose convolution (TConv) is selected to complete upsampling. The parameters inside TConv can be initialized randomly and updated iteratively. At the same time, the SmoothL1Loss(SLL) function is used as the loss function of the denoising autoencoder, which can better measure the error between the estimated value and the real value, reconstruct a more accurate modulation signal, and improve the accuracy of the model training.

The remainder of this paper is organized as follows. Section 2 describes the communication system model. Section 3 analyzes the applicability of the neural network and introduces the principles and details of the proposed DRdA-CA. Section 4 describes the dataset generation and preprocessing. The simulation results and performance analysis are summarized in Section 5. Finally, Section 6 concludes the paper.

## 2. Communication System Model

**Figure 1:**
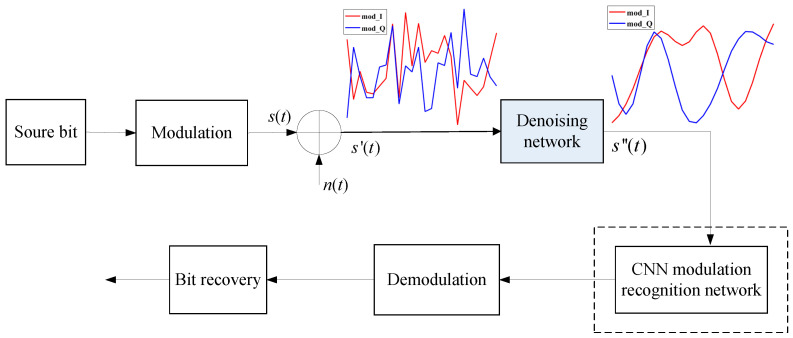
Communication system model.

In this paper, we focus on the communication scenarios under the additive white Gaussian noise (AWGN) channel [33] with a low SNR. The AWGN channel is a widely used channel model between the transmitter and the receiver in wireless communication systems, in which the noise random variable obeys the zero-mean Gaussian distribution. We consider cooperative communication systems and non-cooperative communications systems and the modulation types of the systems are diversified. The system model is shown in Figure 1. At the transmitter, the source bit is modulated and sent to the channel, in which the modulation signal s(t) will be affected by AWGN.

The received modulation signal $s^{\prime }\left(t\right)$ can be expressed as Equation (1).

(1) $$s^{\prime }\left(t\right)=s\left(t\right)+n\left(t\right)$$

where s(t) is the modulation signal with IQ components and generated at transmitter and n(t) represents zero-mean complex AWGN vector with variance $\sigma^{2}$ in each signal dimension. When the noise interference is serious, the received signal quality is very poor, which will badly affect the demodulation performance. In this case, the noise reduction needs to be performed before subsequent processing. In the cooperative communication scenario, the modulation type is known in advance for the receiver, thus the denoised signal will be demodulated directly. For the application of non-cooperative communication, the receiver does not know the current modulation type, thus it needs to firstly perform modulation recognition, as shown in the dashed box in the Figure 1. We chose the automatic modulation classification (AMC) module proposed in [34] to finish the modulation recognition.

This paper focuses on signal denoising based on deep learning, which corresponds to the denoising network module in Figure 1. We will develop a novel network architecture for noise reduction of multiple modulated signals. Based on the above analysis, eight modulated signals, which are in good agreement with the communication system model described in our paper, are generated using MATLAB software simulation.

## 3. DRdA-CA Architecture Details

### 3.1. The Proposed Denoising Autoencoder Structure

**Figure 2:**
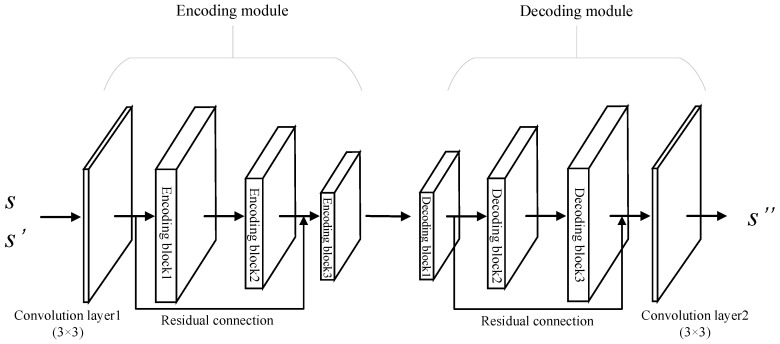
Dual residual denoising autoencoder network framework with channel attention.

This paper proposes a dual residual denoising autoencoder with a channel attention mechanism, and the network model can be seen in Figure 2, which is composed of an encoding module and decoding module in series. The encoding module consists of a 3×3 convolutional layer and three encoding blocks, and each encoding block is the improved SE-ResNet module. Similarly, the decoding module is also composed of a 3×3 convolutional layer and three decoding blocks. It is worth noting that, in the encoding and decoding modules, we further introduce the residual connection between encoding (decoding) blocks at different levels to optimize the network degradation problem.

This paper focuses on the AWGN channel, and the received modulation signal $s^{\prime }\left(t\right)$ can be expressed as Equation (1). The task of the noise reduction autoencoder is to obtain the optimal estimation $s^{\prime \prime }\left(t\right)$ of the pure modulation signal s(t) from the $s^{\prime }\left(t\right)$.

In the decoding modules of the proposed DRdA-CA, the Tanh function defined by Equation (2) is used as the activation function of the convolution layer, which is the output layer.

(2) $$f_{Tanh}\left(x_{i}\right)=\frac{e^{x_{i}}-e^{-x_{i}}}{e^{x_{i}}+e^{-x_{i}}}\;$$

where $x_{i}$ is the input variable. The detailed network parameters of the proposed DRdA-CA are shown in Table 1.

### 3.2. The Encoding and Decoding Blocks

**Figure 3:**
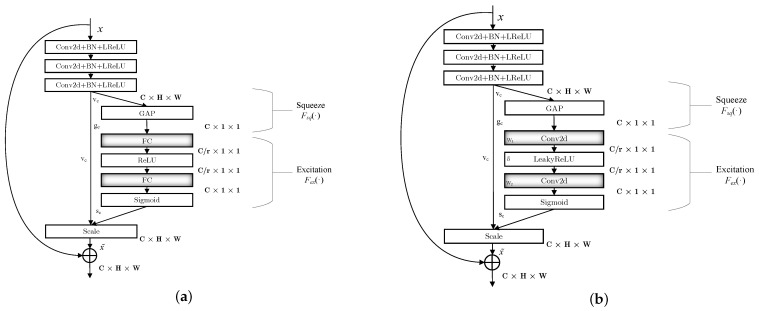
Improved SE-Resnet and Original SE-Resnet modules. “⊕” represents addition operation. (**a**) Original SE-ResNet module. (**b**) Improved SE-ResNet module (encoding block).

The Squeeze-and-Excitation Deep Residual Network (SE-ResNet), as the champion model of Imagenet 2017, is very similar to the Deep Residual Network (ResNet). However, it adds an SE module so that the information between the channels can be exchanged, thereby improving the performance of the network. Figure 3a depicts the original SE-ResNet module. We modify the SE unit in SE-ResNet and use the improved SE-ResNet to establish the encoding and decoding blocks. That is, the fully connected layer (FC) in the original SE unit is replaced by a convolutional layer. This can be attributed as follows. (1) The SE-ResNet was originally designed and implemented under the Caffe framework, in which the FC supports the input of data dimension 1×1×C. However, the deep learning framework Pytorch used in this paper does not support the input of data with the same dimension. When the FC is used in the model, both the input and output data require dimension transformation, i.e., three-dimensional data 1×1×C should be reduced and flattened into one-dimensional data C as the input of the fully connected layer. It will increase the computational complexity of the model and destroy the spatial structure of the data, leading to the loss of feature information. This problem can be addressed by replacing the FC with a 1×1 convolutional layer. (2) Both the convolutional layer and FC perform a dot product operation on the feature maps, thus they have the same functional form. That is, the 1×1 convolutional layer can be used to replace the FC without changing the function operation of the SE module itself. In addition, since the value of the modulation signals is bipolar with positive and negative numbers, we choose LeakyReLU as the activation function. The improved SE module, as shown in Figure 3b, mainly consists of the following two parts.

(1) The squeeze part: Assume that the dimension of the original feature map is C×H×W, where *C* represents the number of channels in the feature map, *H* represents the height of the feature map, and *W* is the width of the feature map. The task of the squeeze part is to compress the feature map dimension C×H×W to C×1×1, which is equivalent to squeezing the matrix in the dimension of of H×W into the matrix in the dimension of 1×1. The squeezed matrix can still obtain the previous global field of view and has a wider perception area.

The squeeze operation is implemented using the Global Average Pooling (GAP) method, and its calculation is described as follows:

(3) $$g_{c}=F_{sq}\left(v_{c}\right)=\frac{1}{H\times W}\;\sum_{i=1}^{H}\sum_{i=1}^{W}v_{c}\left(i,j\right)$$

where $v_{c}$ is the feature map output by the third convolutional layer in the improved SE module. After GAP processing for $v_{c}$, we get the feature map $g_{c}$, and the same below.

(2) The excitation part: By adding a convolutional layer above and below the nonlinear activation layers, we parameterize the gating mechanism to predict the importance of each channel in the feature map $g_{c}$. Then the weight values of different channels are obtained and applied to the original feature map to complete the excitation. The excitation operation process is performed as follows. First, the compressed feature map $g_{c}$ is fed into a convolutional layer with *C*/*r* output channels, namely a dimensionality-reduction layer with reduction ratio *r*. Then the convolution result is activated using the LeakyReLU function, and finally the activated feature graph is dimensionally augmented through a convolution layer with *C* output channels to guarantee channel dimensional consistency between the output $s_{c}$ and feature map $v_{c}$. The excitation function $F_{ex}$ is given by

(4) $$s_{c}=F_{ex}\left(g_{c},W\right)=\sigma \left(W_{2}\delta \left(W_{1}g_{c}\right)\right)$$

where $W_{1}\in R^{\frac{C}{r}\times C}$ and $W_{2}\in R^{C\times \frac{C}{r}}$ are the weight values of the two convolutional layers around the LeakyReLU layer, and δ is the LeakyReLU function with slope parameter α=0.001. The LeakyReLU function solves the problem of neuron necrosis in the ReLU function, and σ represents the sigmoid activation function. Finally, the feature map $s_{c}$ output by the sigmoid function is used to adjust the channel weights of feature map $v_{c}$, and the calculation process is given by

(5) $$\tilde{x}=F_{scale}\left(v_{c},s_{c}\right)=s_{c}v_{c}$$

Based on above analysis, the excitation operation can map the feature map $g_{c}$ to a set of channel weights $v_{c}$.

**Figure 4:**
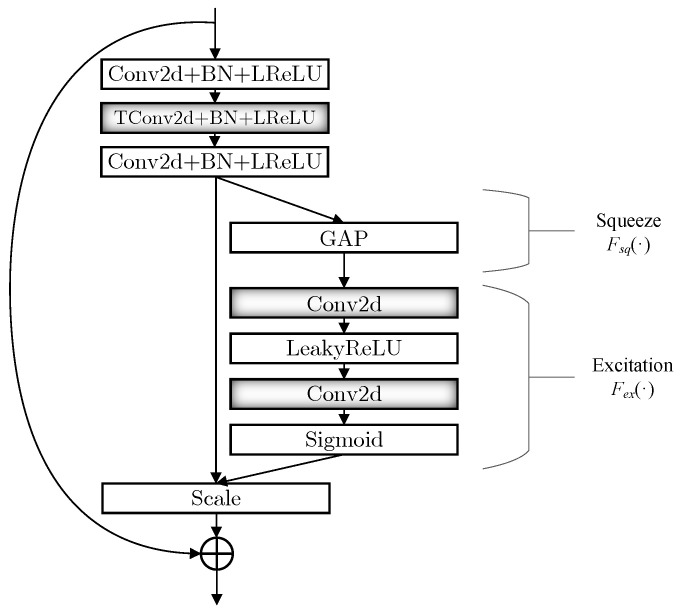
Decoding block.

The decoding block of the noise reduction autoencoder is depicted in the Figure 4. It is clear that the decoding block is similar to the encoding block, but it adopts TConv instead of one of the convolution layers in the original encoding block to realize the upsampling operation. Like the convolution operation, TConv is learnable. The advantage of transpose convolution is that it can theoretically obtain the upsampled value that is most suitable for the current dataset through continuously updating the parameters.

### 3.3. Loss Function

In the existing work, most denoising autoencoders use the two loss functions of Mean Absolute Error (MAE) and Mean Square Error (MSE), and carry out parameter estimation by minimizing the error. MAE is the average absolute value of the true value minus the predicted value. By contrast, MSE is the average square of the absolute value of the predicted value minus the true value. Both MSE and MAE range from 0 to infinity, and they can be calculated, respectively, using

(6) $$MAE=\frac{1}{N}\;\sum_{i=1}^{N}\left|\left(s_{i}-s_{i}^{\prime \prime }\right)\right|$$

(7) $$MSE=\frac{1}{N}\;\sum_{i=1}^{N}{\left|\left(s_{i}-s_{i}^{\prime \prime }\right)\right|}^{2}$$

where *N* is the length of the input signal, $s_{i}$ is the noise-free signal value, and $s_{i}^{\prime \prime }$ is the signal-reconstructed value (predicted value) output from our autoencoder, the same as below. The problem existing in the MAE training process is that the update gradient is always the same. Even for a small loss value, the gradient is relatively large, which is not conducive to a training model. Unlike MAE, MSE will assign more weight to outliers, and, at the expense of the error of other samples, the model will be updated towards reducing the error of the outliers, which will result in the performance degradation of the model. In order to overcome the above defects and make the output of the denoising autoencoder more accurate, the SLL is adopted in the proposed DRdA-CA in this paper. The SLL is calculated as follows:

(8) $$SLL\left(s,s^{\prime \prime }\right)=\frac{1}{N}\sum_{i=1}^{N}z_{i}$$

where the variable $z_{i}$ is defined as follows:

(9) $$z_{i}=\left\{\begin{array}{c} 0.5\times |s_{i}-s_{i}^{\prime \prime }{|}^{2},\mathrm{if}\left|s_{i}-s_{i}^{\prime \prime }\right|<1\\ |s_{i}-s_{i}^{\prime \prime }|-0.5,\mathrm{otherwise} \end{array}\right.$$

It is clear that SLL is an integration of MSE and MAE. When taking the derivative of SLL, we have:

(10) $$\frac{dSSL}{ds_{i}}\;=\left\{\begin{array}{c} s_{i},\mathrm{if}\left|s_{i}\right|<1\\ \pm 1,\mathrm{otherwise} \end{array}\right.$$

In Equation (10), it can be seen that, when $s_{i}$ is small, the derivative of SSL with respect to $s_{i}$ also becomes small. Otherwise, the absolute value of the derivative reaches the upper bound of 1, and the network stability will not be impacted by the large derivative. As we know, MSE has derivatives at the origin and is easy to converge, and, in the boundary area, MAE enables our denoising autoecoder to correct back when the error tends to be large. Regarding the two error metrics, therefore, the SLL is more robust with respect to outliers.

## 4. Dataset

### 4.1. Experimental Data and Environment

The dataset of the denoising autoencoder includes eight modulation signals generated by MATLAB; they are BPSK, QPSK, 8PSK, CPFSK, GMSK, OQPSK, 16QAM, and 64QAM, and the detailed parameters of the dataset are shown in Table 2. The SNR of the modulation signals ranges from −12 dB to 8 dB, and the SNR interval is 2 dB. In the transmission process of the signal, the transmitter completes the modulation and up-conversion of the signal, and then transmits radio frequency signals into the channel. The receiver first performs down-conversion, and subsequently estimates the carrier frequency and phase, restores the received signal to the baseband signal, and performs modulation recognition, demodulation, and other operations. Without the loss of generality, this paper takes the baseband signal as an example to generate the dataset of the modulation signals. The rate of the baseband signal is set to 256 kHz, and the sampling frequency of the signal is 1024 kHz—that is, the oversampling rate of the signal is 4 times higher to increase the fault tolerance rate of the signal. The modulated signals are shaped and filtered using a root raised cosine filter with a roll-off factor of 0.3, and the transmission channel is modelled as an AWGN channel. Pytorch is used as the deep learning framework, and an NVIDIA GeForce RTX2080 Ti graphics card is used to implement the GPU parallel acceleration calculation.

### 4.2. Dataset Preprocessing

In order to facilitate the addition of noise and the measurement of the SNR, as well as to accelerate the training of the model and reduce the computational complexity of the data, we normalize the power of the modulation signals generated by MATLAB simulation using the following formulae:

(11) $$P_{s}=\frac{\sum_{i=1}^{N}{\left|s_{i}\right|}^{2}}{N}\;$$

(12) $$N_{s}=\frac{S}{\sqrt{P_{s}}}\;$$

where $s_{i}$ is the initial noise-free modulation signal, *N* is the length of the signal, $P_{s}$ is the signal power, and $N_{s}$ is the noise-free modulation signal with normalized power. In the training stage of the denoising autoencoder, the signal dataset with 11 different SNR values is made into an IQ data sample with the shape of 2 × 1024 for each piece. We split the training set and the values set by the ratio of approximately 8:2, and there are 70,400 pieces of training data and 17,600 pieces of validation data. Then we generate 8800 pieces of test data under the same parameters. The Adaptive Moment Estimation (Adam) optimization algorithm is used to complete model training with 40 training epochs, and the initial learning rate is set to 0.001 while decreasing by 0.1 times every 20 epochs.

## 5. Experiment and Analysis

### 5.1. Layer Number Optimization

We conduct simulation experiments to evaluate our denoising autoencoder, and we also compare it with a set of typical models including RLS, LMS, PCA, and the deep learning method DnCNN proposed by Khan et al. [18]. The evaluation metrics are SNR, Error Vector Magnitude (EVM), and demodulation BER before and after noise reduction. In addition, the modulation recognition accuracy is considered and the modulation recognition model is the CNN [34].

**Figure 5:**
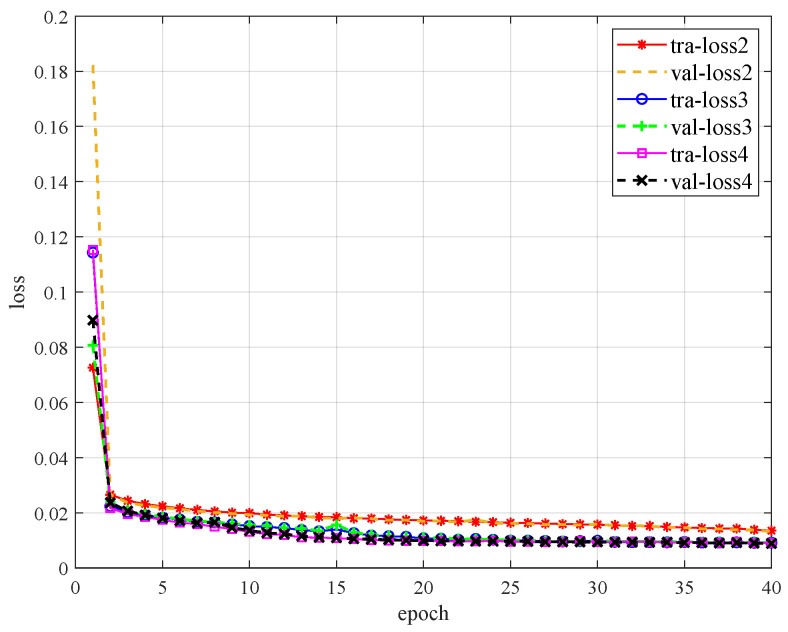
Classification accuracy at different depths.

The SNR is defined as the ratio of noiseless signal power to noise power, which can intuitively measure the quality of signal. The calculation formula is given by the following:

(13) $$SNR_{dB}=10\mathrm{lg}\left(\frac{{\left||s|\right|}_{2}^{2}}{\left|\right|s^{\prime }-s{\left|\right|}_{2}^{2}}\;\right)$$

where *s* represents the noiseless modulation signal, and $s^{\prime }$ represents the noisy modulation signal. The parameter EVM can comprehensively measure the amplitude error and phase error of the modulation signal, which is defined as the ratio of the root mean square value of the error vector signal and the root mean square value of the ideal signal, and expressed in the form of a percentage. The calculation formula is given by the following:

(14) $$EVM=\sqrt{\frac{\frac{1}{N}\sum_{i=1}^{N}|s_{i}-s_{i}^{\prime \prime }{|}^{2}}{\frac{1}{N}\sum_{i=1}^{N}|s_{i}{|}^{2}}}\times 100\%$$

where *s* is the same as before, and $s_{i}^{\prime \prime }$ represents the signal-reconstructed value (predicted value). In the first experiment, we determine the optimal depth of encoder-decoder modules. We design three encoder module structures, whose encoder-decoder depths equal 2, 3, and 4, respectively, and the corresponding neural networks are called DRdA-CA2, DRdA-CA3, and DRdA-CA4, respectively. The training loss and validation loss of the three network models are shown in Figure 5. The detailed value of the training loss with different epochs and the numbers of parameters are listed in Table 3. In terms of the training loss, DRdA-CA3 is 1.4–1.9% lower than DRdA-CA2 on average when the epoch is larger than 10, and the validation loss presents a similar trend. It should be noted that the performance of DRdA-CA3 and DRdA-CA4 is almost the same. When it comes to the complexity, the size of the model parameters of DRdA-CA2, DRdA-CA3, and DRdA-CA4 are 181 k, 263 k, and 345 k, respectively. Therefore, considering both the loss and model complexity, we choose DRdA-CA3 as the neural network model structure for the denoising tasks. Although the loss values have been small at the epoch equaling 10, they continue to decline and basically stabilize at 40 rounds. In this paper, the model parameters adopt the training results of 40 rounds.

### 5.2. Ablation Experiments

**Figure 6:**
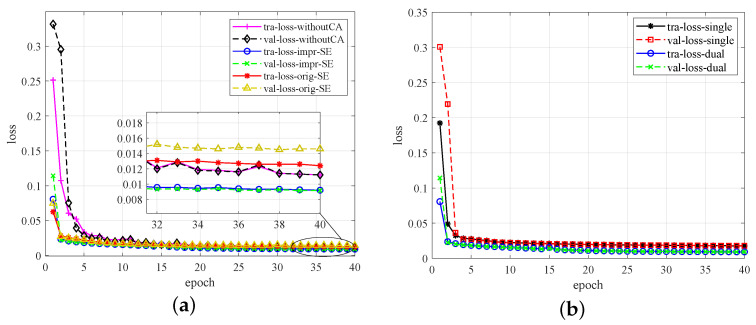
Ablation experiments. (**a**) Original SE and improved SE. (**b**) Single and dual residual connections.

In this paper, the SE module is first improved and then introduced into the encoder as the channel attention module, so that the modulation signal features can be better extracted and the computational complexity of the model can be reduced. In order to validate the efficiency of the improved SE module as the channel attention mechanism, we carry out the first ablation experiment based on the control variable method. We compare the improved SE module (impr-SE) using the convolutional layer with the original SE module (orig-SE) using FC and the denoising autoencoder without a channel attention mechanism (withoutCA). In this case, the SE module is removed in the coding block in Figure 3b. Figure 6a displays the training loss (tra-loss) and validation loss (val-loss) of the three methods. As is shown, the module with the improved SE module gives better performance. When the epoch equals 40, it lower the validation loss by 35.7% and 17.9% compared to the module with the original SE and the model without a channel attention mechanism. In addition, it should be noted that the denoising model without a channel attention mechanism is even superior to the model with the original SE. This can be attributed to the loss of feature information caused by data dimension transformation. Therefore, a mismatched channel attention module may cause network performance degradation, and the improved SE is quite effective.

In the proposed DRdA-CA, “dual” represents the residual connections added not only between different encoding (decoding) blocks but also inside the encoding (decoding) blocks of different depths, so it is called “dual”. In contrast, “single” refers to the residual connection only existing inside the encoding (decoding) block, and each encoding (decoding) block is the improved SE-ResNet module, which contains one residual connection. In order to demonstrate the advantages of “dual residual connections”, one of the main innovations, we have also conducted an ablation experiment based on the control variable method, comparing it with the “single residual connection” method, where the residual connection only exists inside the encoding (decoding) block. The loss curves are given in Figure 6b. As shown in the figure, the dual residual model achieves a lower training loss and validation loss than the single residual model. When the epoch increases to 40, the validation loss of the dual residual connections is only 50% of that of the single residual connection. Comparing Figure 6a,b, apparently, both the improved SE module and dual residual connections play an important role in improving denoising performance, and dual residual connections are more effective.

### 5.3. Comparison of SNR Improvement

In the second experiment, we compare the SNR and EVM variations of different denoising methods. Table 4 displays the average values of SNR improvement in overall SNR, ranging from −12 dB to 8 dB for all eight modulation modes with five different denoising methods. In the SNR range of −12 dB to 8 dB, taking the GMSK signal as an example, the SNRs of the modulation signal are increased by 6.76 dB, 6.5 dB, and 7.18 dB, respectively, on average, after denoising with RLS, LMS, and PCA algorithms. When the DnCNN model is used for noise reduction, the SNRs of the signal are improved by 11.59 dB on average. Remarkably, the proposed DRdA-CA brings an increase of 16.8 dB on average in terms of the SNRs. When DRdA-CA is applied to 8PSK, 16QAM, and 64QAM signals, the average values of SNR enhancement are 9.47 dB, 8.38 dB, and 8.27 dB, respectively. For two deep learning-based denoising methods, DnCNN and DRdA-CA, the average values of SNR improvement gradually go up with the decrease in modulation order, except in a few cases. However, traditional noise reduction methods do not show similar laws. It is clear that DRdA-CA outperforms the DnCNN method and RLS, LMS, and PCA. This can be attributed to the well-designed structure in the proposed method, in which both double-residual connections and the introduction and improvement of the SE module contribute to a desirable denoising effect.

**Figure 7:**
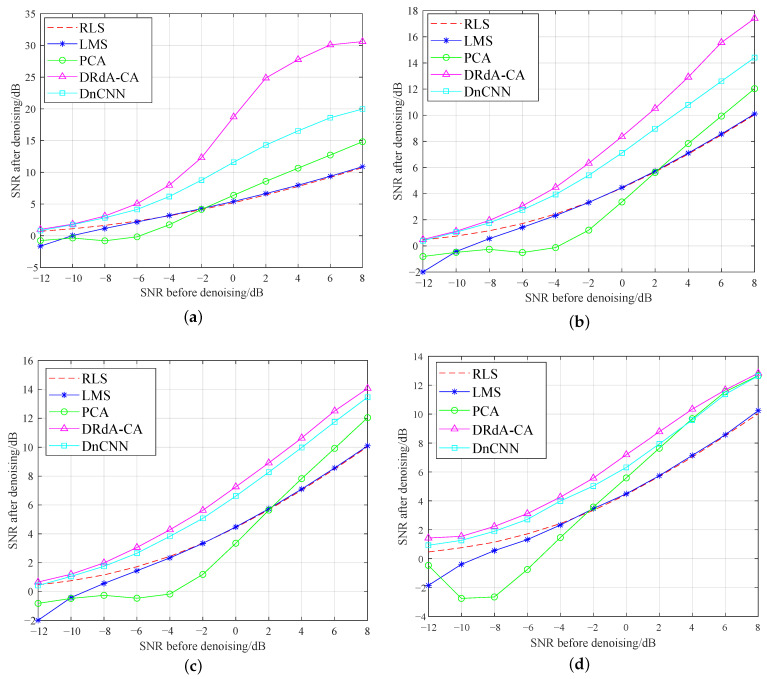
SNR of modulation signal after denoising [2,3,5,6,7,18]. (**a**) GMSK. (**b**) 8PSK. (**c**) 16QAM. (**d**) 64QAM.

**Figure 8:**
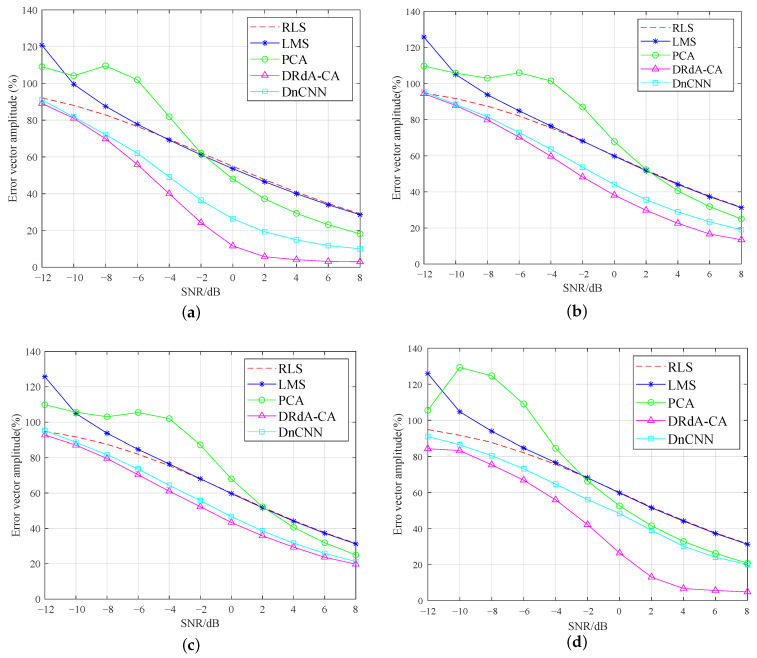
EVM after denoising of modulation signal [2,3,5,6,7,18]. (**a**) GMSK. (**b**) 8PSK. (**c**) 16QAM. (**d**) 64QAM.

It would be complicated to use curves to display dynamic ranges of SNR and EVM after denoising with the increase of SNR for all eight modulation modes. As a result, we only present four typical modulation modes in Figure 7 and Figure 8. Figure 7a–d respectively show the SNR variations of GMSK, 8PSK, 16QAM, and 64QAM, and four signals are processed using different denoising methods. It can be seen from Figure 7 that the deep learning denoising methods, denoted as DRdA-CA and DnCNN, are superior to the three traditional denoising methods, denoted as LMS, RLS, and PCA. The proposed denoising method achieves different denoising effects for different modulation modes. Although denoising is more challenging for high-order modulation due to the complex signal patterns, the proposed denoising achieves a desirable denoising performance for high-order modulation. Moreover, for the four modulation types, the proposed DRdA-CA has always maintained the maximum SNR improvement over the entire SNR range.

Figure 8a–d also show the EVM curves of the above four modulation signals processed using different noise reduction methods. The lower the EVM, the better the noise reduction performance. As seen from Figure 8, the EVMs obtained using the two deep learning-based denoising methods are significantly lower than those obtained using the other three traditional denoising methods. After PCA denoising, the EVMs of the signals do not steadily decline with the increase in the SNR, which indicates that the robustness of PCA is poor. The signal EVM curves obtained after RLS and LMS denoising are close to each other when the SNR is greater than 4 dB. However, when the SNR falls in −12 dB∼4 dB, RLS is better than LMS. The deep learning algorithm DnCNN performs better than the three traditional algorithms do, and the proposed DRdA-CA makes a better achievement in denoising than DnCNN does.

### 5.4. Comparison of AMC Performance

For non-cooperative communication applications, the receiver needs to firstly perform modulation classification, followed by demodulation and decoding. Therefore, the modulation classification experiment is also done to further verify the performance of the denoising autoencoder. The LMS and RLS algorithms need to know the modulation mode and send the training sequence when they are used to reduce noise in communication systems, leading to low transmission efficiency. Thus, they are unsuitable for non-cooperative communication applications. PCA also has the lowest denoising performance. As a result, in the experiment of the noise reduction cascade AMC, we compare the DnCNN to the proposed method. Moreover, the CNN [34] is adopted to implement AMC, and the dataset is given in Section 4.1, including eight modulation signals under eleven SNRs.

**Figure 9:**
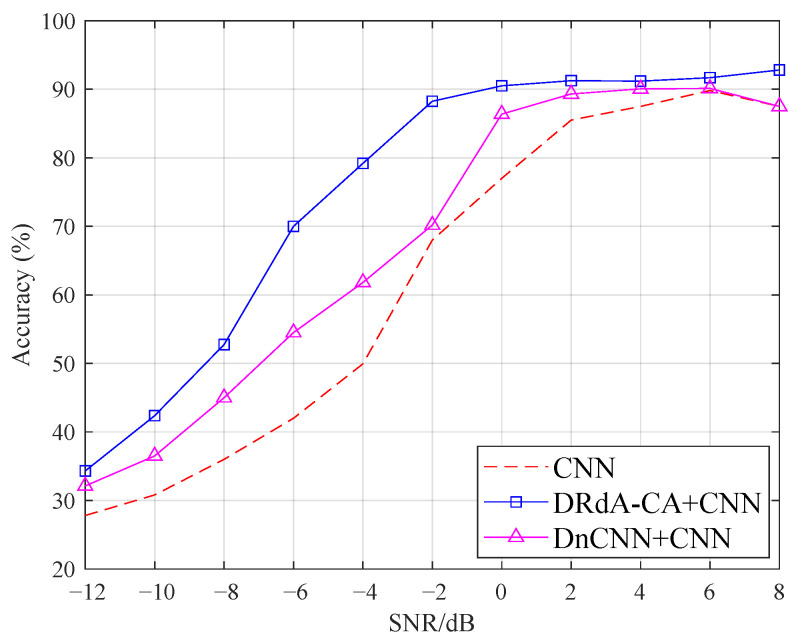
Modulation classification accuracy under different deep learning networks.

The experiment schemes are as follows. (1) The CNN model is used for AMC directly, denoted as CNN. (2) The DnCNN method is used to reduce the noise of modulation signals, and then CNN is used for AMC, denoted as DnCNN+CNN. (3) The proposed DRdA-CA is used to decrease the noise, followed by CNN for AMC, denoted as DRdA-CA+CNN. Figure 9 displays the average classification accuracy of all the modulation modes for three different schemes. The modulation recognition accuracy is greatly increased when using denoising methods based on deep learning. The DRdA-CA method clearly outperforms the DnCNN method. When the SNR varies from −12 dB to 8 dB, compared with the non-denoising scheme, the scheme with DnCNN denoising improves the average classification accuracy by 0.01%∼12.5%. After using the proposed denoising approach, the average classification accuracy is increased by 1.88%∼29.25%. Compared to the DnCNN denoising method, the proposed DRdA-CA makes the average classification accuracy increase from 67.59%∼74.94% over the entire SNR range, which further shows the superiority of the proposed method.

**Figure 10:**
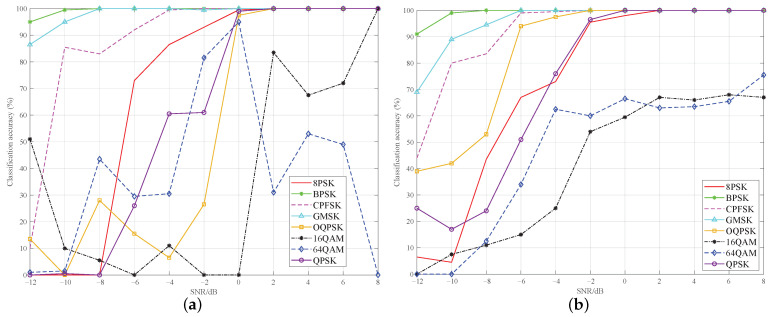
Classification accuracy of different networks for each modulation type. (**a**) DnCNN+CNN. (**b**) DRdA-CA+CNN.

Figure 10a,b give the recognition accuracy of each modulation type with the latter two schemes. As seen in Figure 10a, after DnCNN denoising, the recognition accuracy of the three low-order modulations, BPSK, GMSK, and CPFSK, is relatively high, and almost reaches 100% under the SNR of −4 dB. This is because these three types are low-order modulations and the patterns of the signals are simple and significantly different from those of the high-order PSK and QAM. Therefore, their classification accuracy is also high, even through general DnCNN denoising.

However, the recognition performance of the high-order modulations is poor. In particular, the recognition accuracy of the 16QAM and 64QAM signals fluctuates widely. This is attributed to the highly similar characteristics of higher-order modulation signals and the insufficient noise reduction capability of DnCNN. It should be noted that the classification accuracy of QPSK and OQPSK is lower than that of 8PSK in most of the SNR intervals, although their modulation order is smaller than that of 8PSK. This is because the QPSK and OQPSK signal patterns are too similar, and they are very easy to confuse when the noise reduction effect is limited with the DnCNN method.

According to the Figure 10b, DRdA-CA denoising makes the recognition accuracy of each modulation signal increase significantly. Although 16QAM and 64QAM still cannot achieve accurate classification when the SNR is larger than 2 dB, their recognition accuracy exhibits a continuous rising trend with an increase in the SNR. It demonstrates that the strong denoising ability of the proposed DRdA-CA is beneficial for AMC. The classification accuracy of 16QAM and 64QAM can be further improved by adopting better modulation classification model after denoising, and this paper focuses on the design of the denoising network.

### 5.5. Comparison of Demodulation Performance

**Figure 11:**
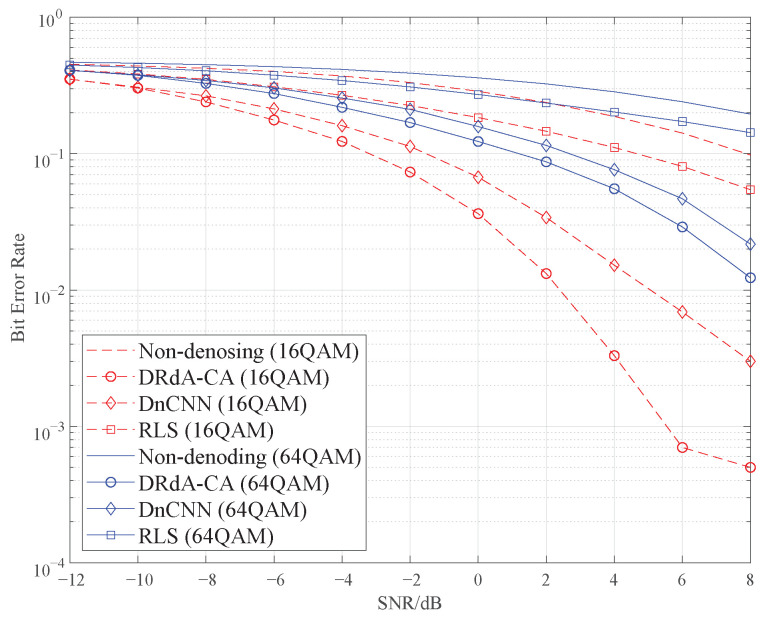
Demodulation BER after denoising.

In order to further verify the performance of the proposed DRdA-CA, the demodulation experiments after denoising have also been done. We present two higher-order modulation modes, 16QAM and 64QAM, since their demodulations are more challenging in the low SNR range for practical communication systems. The RLS algorithm with better performance in the traditional methods and the DnCNN are selected as the comparison methods. Figure 11 shows the BER curves of demodulation for the 16QAM and 64QAM signals after denoising with different methods.

From Figure 11, it can be seen that the three noise reduction methods can improve the BER performance of signal demodulation. The DnCNN improves BER more than the traditional RLS, and the proposed DRdA-CA considerably outperforms both of these two methods. When the SNR ranges from −12 dB to 8 dB, the proposed method decreases the BER by 22.3%∼99.5% for 16QAM and 12.6%∼93.7% for 64QAM compared to the non-denoising method. In addition, compared with RLS and DnCNN, DRdA-CA reduces the BER of 16QAM by 63.5% and 40.5% on average, and makes the BER of 64QAM decline by 46.7% and 18.6% on average, across the entire SNR range. For 16QAM signals at an SNR of 8 dB, when using the RLS and DRdA-CA, the BER of demodulation is reduced by two orders of magnitude, from 0.05 to 0.0005. Besides, compared with DnCNN, the BER of DRdA-CA is only 1/6 of the former. When it comes to the 64QAM signals at an SNR of 8 dB, compared with the non-denoising method, DRdA-CA makes BER decrease from 0.195 to 0.0123, and compared to the RLS and the DnCNN, it reduces the BER of 64QAM by 91.49% and 43.34%, respectively.

## 6. Conclusions

In this paper, a dual residual denoising autoencoder with channel attention (DRdA-CA) is designed based on CNN to deal with the problems caused by a low SNR in wireless communications. First, as a channel attention mechanism, the SE module with one residual connection is adapted and applied to the proposed DRdA-CA to improve the feature extraction ability of neural networks. Then, the other residual connection between the different coding (decoding) blocks is introduced to solve the network degradation problem, which promotes the fusion of model characteristic information at different depths, and further enhances the network noise reduction performance. Moreover, a dataset including eight modulation types is created under the AWGN channel model.

The ablation experiments are done to verify the efficiency of the improved SE module and the dual residual connections, and the dual residual connections are more helpful in improving the denoising performance. Then, the experiments on SNR improvement, modulation classification, and demodulation are also done to verify the advantage of the proposed method. The simulation results show that the proposed DRdA-CA surpasses the traditional denoising algorithms and the DnCNN noise reduction method in both improving the SNR and recovering the original signals for all eight modulation modes. Taking GMSK and 8PSK as examples, the proposed DRdA-CA improves the SNR by 16.84 dB and 9.47 dB on average, respectively. Compared to the DnCNN denoising method, the proposed DRdA-CA makes the recognition accuracy of each modulation signal increase significantly. Even the recognition accuracy of 16QAM and 64QAM goes up steadily with the increase in the SNR. Besides, demodulation performance is also dramatically improved after using the proposed DRdA-CA to reduce noise. Taking the SNR of 8 dB as an example, for 64QAM signals, the proposed DRdA-CA lowers BER by 43.34% compared to the DnCNN method. These experiments further demonstrate our method’s strength. In the future, we will extend our designs and make it suitable for the fading-channel scenario, where modulation signals are prone to be affected by multipath propagation and the Doppler frequency shift.

## Figures and Tables

**Table 1:** Denoising Autoencoder’s Parameters.

Layer Name	Output Feature Map Size
convolutional layer 1	32 × 2 × 1024
encoding block 1	32 × 2 × 1024
encoding block 2	64 × 2 × 512
encoding block 3	128 × 1 × 256
decoding block 1	128 × 2 × 521
decoding block 2	64 × 2 × 1024
decoding block 3	32 × 2 × 1024
convolutional layer 2	1 × 2 × 1024

**Table 2:** Parameter settings for data sample sets.

Parameter	Value/Description
SNR	−12:2:8 (dB)
Symbol rate	256k Baud
Sample rate	1024 kHz
Oversampling rate	4
Forming filters	Root raised cosine filter
Roll-off factor	0.3
Channel	AWGN channel

**Table 3:** The Parameters of Different Depths.

Model	Parameter
DRdA-CA2	181,408
DRdA-CA3	263,416
DRdA-CA4	345,424

**Table 4:** Average value of SNR improvement under different denoising methods.

	RLS	LMS	PCA	DnCNN	DRdA-CA
GMSK	6.76 dB	6.50 dB	7.18 dB	11.59 dB	16.84 dB
CPFSK	6.14 dB	5.75 dB	8.45 dB	10.11 dB	15.39 dB
BPSK	6.15 dB	5.74 dB	8.46 dB	11.12 dB	11.86 dB
QPSK	6.14 dB	5.74 dB	6.21 dB	9.17 dB	13.44 dB
OQPSK	6.14 dB	5.76 dB	6.17 dB	8.99 dB	13.43 dB
8PSK	6.13 dB	5.74 dB	5.44 dB	8.29 dB	9.47 dB
16QAM	6.13 dB	5.75 dB	5.43 dB	7.9 dB	8.38 dB
64QAM	6.14 dB	5.78 dB	6.19 dB	7.79 dB	8.27 dB

## Data Availability

The dataset is available from the website https://github.com/GJX2810/moddataset.git (accessed on 14 November 2022).

## Abbreviations

The following abbreviations are used in this manuscript:

BER	Bit Error Rate
SNR	Signal-to-Noise Ratio
DRdA-CA	Double-Residual Denoising Autoencoder Method with Channel Attention Mechanism
WT	Wavelet Transform
LMS	Least Mean Square
RLS	Recursive Least Squares
PCA	Principal Component Analysis
SVD	Singular Value Decomposition
CNN	Convolutional Neural Network
FC	Fully Connected Layers
ML	Machine Learning
VKF	Vector Kalman Filter
SCM	Spatial Channel Model
DnCNN	Residual Learning of Deep CNN for Image Denoising
SE	Squeeze-and-Excitation
TConv	Transpose Convolution
SLL	SmoothL1Loss
AWGN	Additive White Gaussin Noise
AMC	Automatic Modulation Classification
SE-ResNet	Squeeze-and-Excitation Deep Residual Networks
ResNet	Deep Residual Network
GAP	Global Average Pooling
MAE	Mean Absolute Error
MSE	Mean Square Error
Adam	Adaptive Moment Estimation
EVM	Error Vector Magnitude
